# Thyroid Regeneration: How Stem Cells Play a Role?

**DOI:** 10.3389/fendo.2014.00055

**Published:** 2014-04-14

**Authors:** Shioko Kimura

**Affiliations:** ^1^Laboratory of Metabolism, National Cancer Institute, National Institutes of Health, Bethesda, MD, USA

**Keywords:** adult-resident thyroid stem/progenitor cells, partial thyroidectomy, solid cell nest, ultimobranchial body cyst, side population, sphere/spheroid culture, OCT4 expression

## Abstract

Many tissues if not all are thought to contain stem cells that are responsible for regeneration and repair of the tissue after injury. Dysregulation of tissue regeneration may result in various pathological conditions, among which cancer is the most extensively studied. Notably, the so-called cancer stem cells or tumor-initiating cells, have been studied in order to understand the mechanisms of carcinogenesis and/or metastasis. However, the nature of cancer stem cells, let alone normal stem/progenitor cells, particularly those of the thyroid remains elusive. There remains a gap in knowledge between adult thyroid stem/progenitor cells and cancer stem cells of the thyroid, and if and/or how they are related to each other. Understanding of the mechanism for thyroid regeneration and mode of participation of normal adult thyroid stem/progenitor cells in this process will hopefully yield a more complete understanding of the nature of thyroid cancer stem cells, and/or help understand the pathogenesis of other thyroid diseases. This review summarizes the current understanding of adult thyroid stem/progenitor cells, with particular emphasis on how they contribute to thyroid regeneration.

## Introduction

Stem cells can be categorized in three groups; embryonic stem cells, adult tissue stem/progenitor cells, and cancer stem cells. Only in recent years, research on all categories of stem cells in the thyroid field has begun to emerge. Lin et al. for the first time reported the differentiation of mouse embryonic stem cells into thyrocyte-like cells *in vitro* (1). Several *in vitro* studies followed examining the effect of TSH, insulin, insulin-like growth factor 1 (IGF1), and/or activin A on differentiation and/or maturation of embryonic stem cells into thyrocytes (2–6). In 2012 (7), functional thyroid follicles were successfully generated *in vitro* from mouse embryonic stem cells that over express NKX2-1 (also called TTF1) and PAX8, two transcription factors critical for thyroid development (8, 9) and thyroid-specific expression of genes such as those encoding thyroglobulin (TG), thyroid peroxidase (TPO), TSH receptor (TSHR), and sodium-iodide symporter (NIS) (10–17). These *in vitro*-derived follicles functionally rescued experimentally induced hypothyroidism *in vivo* (7).

For the past several years, a number of studies have characterized adult normal thyroid stem/progenitor cells and thyroid cancer stem cells, the latter using various human thyroid tumors and tumor cell lines to determine the mechanisms of thyroid carcinogenesis and/or metastasis (18–22). Yet the nature of thyroid cancer stem cells is poorly understood. For instance, it is not known whether cancer stem cells are the result of thyroid stem cells acquiring mutations or through epithelial–mesenchymal transition, or a small portion of cancer cells acquiring properties of stem cells following dedifferentiation or through other mechanisms (23–25). Alternatively, fetal thyroid cell carcinogenesis theory suggests that cancer cells are directly generated from fetal cells (26). In order to address these unresolved questions and to understand the nature and/or role of cancer stem cells in thyroid carcinogenesis and/or metastasis, characterization of adult normal thyroid stem/progenitor cells, if present, is of great importance. Our understanding of adult normal thyroid stem/progenitor cells may yield a better understanding of various other thyroid diseases such as thyroiditis in which thyroid tissue stem/progenitor cells may become activated to repair the damage.

## Presence of Adult-Resident Thyroid Stem/Progenitor Cells

Thyroid is an organ of slow turnover, estimated to divide only five times in adulthood (27, 28). The thyroid retains its size and function that are under control of the physiological negative feedback mechanism (27). Following hemithyroidectomy, the residual thyroid tissue undergoes considerable increase in weight due to hypertrophy rather than hyperplasia (29). However, in the case of subtotal thyroidectomy, the presence of hyperplasia was noted (30), suggesting cell proliferation. The presence of a population of stem cells that can respond to such proliferation stimulus *in vivo* was first postulated in early 1990s (27). This hypothesis was mainly based on the earlier experimental results showing that thyroid cells when grafted into thyroidectomized recipient animals, developed functional thyroid follicles, and the number of follicles formed or the thyroid function as determined by T3, T4, and TSH levels, had a direct correlation with the number of cells injected (31–33). In some of these grafts, thyroid neoplasms including undifferentiated thyroid carcinomas were observed after 10–13 months of grafts when cells were irradiated while same number of unirradiated cells produced adenomas in 17–22 months (31). These results were suggestive of the presence of cancer stem cells. Based on this type of experiment and the fact that foci formation in radiobiological cloning experiments occurred at a very low efficiency, the frequency of stem cells was estimated at most as 1 in 1000 (27).

### Side population cells

An attempt to isolate cells having stem/progenitor characteristics was first carried out using side population (SP) cells of mouse thyroid (34). SP cells were originally identified in the adult mouse bone marrow as a small subset of cells able to efflux the vital dye Hoechst 33342 in flow cytometric analysis (35). It was later demonstrated that this was based on the expression of the ATP binding cassette (ABC) family of transporter proteins in stem/progenitor cells, which are able to pump out the dye, thus rendering the dye efflux of SP cells sensitive to inhibitors such as verapamil and fumitremorgin (36, 37). Various non-hematopoietic adult tissues were subsequently demonstrated to possess SP cells, including the liver, skeletal muscle, lung, kidney, and mammary gland (38–45). SP cells isolated from adult muscle or liver contributed to tissue regeneration (40, 44), demonstrating that SP cells contain cells characteristics of stem cells.

Mouse thyroid SP cells were obtained, ranging from 0.3 to 1.4% of the total population of cells. They were composed of two groups of cells characterized as CD45(−)/c-kit (−)/SCA1(+) and CD45(−)/c-kit(−)/SCA1(−), each representing approximately a half of SP cells (34). SCA1 is known to be expressed in SP cells and/or enriched progenitor cell population from various tissues such as bone marrow, mammary gland, and muscle (35, 42, 44, 45). Thyroid SP cells expressed stem cell marker genes such as nucleostemin and *Oct4* in addition to *Abcg2* through which the SP fraction of cells were isolated, while thyroid differentiation marker genes such as *Tg*, *Tpo*, *Tshr*, and two transcription factors critical for thyroid development and thyroid-specific gene expression, *Nkx2-1* and *Pax8* were expressed only in non-SP fraction of cells as determined by qRT-PCR (34). When SP cells were cultured in three-dimensional (3D) collagen gel in media containing fetal calf serum (FBS) *in vitro*, they remained at similar numbers for 9 weeks without significant changes in morphology, while non-SP cells rapidly expanded and spherical structures began to form at around 3 weeks, which further increased in number and size by 5 weeks (34). The fact that SP cells remained viable for 9 weeks without any sign of cell death suggested that they may have stem/progenitor ability, but require correct signal to start proliferation. Further, histological analysis and immunohistochemistry of cultured non-SP cells carried out at 9 weeks demonstrated the presence of follicle-like structures positive for NKX2-1 and TG. These results revealed that mouse thyroid SP cells exhibit stem/progenitor cell-like characteristics.

Side population cells were also found in various human thyroid cancer cell lines (18). They showed clonogenic ability higher than non-SP cells *in vitro*, however in *in vivo* tumorigenesis assays using nude mice, tumors were formed in most cases regardless of cell types. These results suggested that cancer stem cells may not be identical with cells contained in SP cells obtained from wild-type mouse thyroids.

### OCT4-expressing cells

Monolayer cultures derived from human nodular goiters in media containing FBS expressed mRNAs encoding stem cell marker OCT4 and endoderm markers GATA4 and HNF4α for at least seven passages (46). Addition of TSH to the media did not change the percentage of OCT4-positive cells, and no cells were double-positive for OCT4 and GATA4 or HNF4α as judged by flow cytometric analysis. By use of immunostaining of monolayer cultures and human thyroid goiter tissues, a very small number of single isolated cells were present that were positive for OCT4, GATA4, or HNF4α protein. However, qRT-PCR analysis of FACS-isolated GATA4 or HNF4α-positive fraction of cells, or OCT4-positive cells demonstrated the expression of mRNA for OCT4, or the endodermal markers, respectively, suggesting heterogeneous population of *OCT4*-positive cells; some cells may have started commitment toward endoderm lineage. The results thus provided the evidence for the presence of adult stem/progenitor cells of endodermal origin in human thyroid gland (46).

### Thyroid primary sphere culture

Side population cells were isolated from human goiters that express *OCT4* and *ABCG2*, but not other genes *HNF4α*, *GATA4*, *PAX8*, and *TG*, *TPO*, *TSHR*, and *NIS* as thyroid differentiation markers (47). When maintained in monolayer culture or in Matrigel under serum- and TSH-containing medium for up to 14 days, neither cell attachment nor growth was observed even under intense growth stimulation. This situation was somewhat similar to mouse SP cell cultures as described above, in which stem/progenitor-like cells remained non-proliferative in monolayer or 3D cultures for 9 weeks (34).

Single cell suspension of primary human thyrocytes from nodular goiters was cultured in a special serum or TSH free-medium that allowed formation of floating spherical colonies (47). These spheres grew in size during first 3–4 days in culture, and expressed stem cell markers *OCT4* and *ABCG2*, and endodermal markers *GATA4* and *HNF4α*. The sphere-derived cells contained SP cells enriched about 50-fold as compared with those before sphere formation (5 vs. 0.1%, respectively). After 3 days of differentiation initiation with serum, sphere-derived cells began to express *TSHR* gene, which further intensified in the presence of both serum and TSH. These sphere-derived cells differentiated into thyrocytes by day 21, which expressed thyroid differentiation markers, *PAX8*, *TG*, *NIS*, *TSHR*, and *TPO* mRNA, but not stem cells or endodermal markers. Collagen embedded sphere-derived differentiated cells formed thyroid follicle-like structures, which displayed TSH-dependent ^125^iodide uptake, a hallmark of differentiated thyroid cells.

### Thyrospheres-derived cell lines

Thyroid cell aggregates called “thyrospheres” were generated by culturing fresh surgical human thyroid fragments residual to collagenase digestion in defined media containing EGF and bFGF (48). Normal thyroid, normal perinodular tissue of the thyroid adenomas as well as all the different pathological thyroids were used to generate thyrospheres, from which 23 lines were established after 2 months of culture. Twelve such cell lines examined contained a subpopulation of CD34(+)/CD45(−) cells. When these spheroid cells were seeded in collagen gels in the presence of “differentiation medium” containing serum, follicle-like structures were formed. In contrast, spheroids cultured in “spheroid culture medium,” follicle-like structures did not form (48). Cells within the follicles were all positive for TG and the levels of T4 progressively increased in the supernatant of the spheroids cultured in “differentiation medium.” Further, when most freshly isolated spheroids were co-cultured with a neuroblastoma cell line or in adipogenic medium, the produced progeny had the expression of the neuronal marker β-tubulin III or positive staining for oil red O. These experiments demonstrated that stem/progenitor cells exist within the thyroid with an intrinsic ability to generate thyroidal cells and the potential to produce non-thyroidal cells.

All the studies as described provide clear evidence that adult thyroid indeed possesses stem/progenitor cells although the real origin of these cells has yet to be determined. Further, spheres/spheroids formation in serum-free media seems to be critical as the first step to have isolated stem/progenitor cells differentiate into functional thyrocytes *in vitro*. Indeed, when mouse and human embryonic stem cells were subjected to differentiation into thyrocytes in serum-free or growth factor-reduced medium, they went through embryoid bodies, similar in shape as spheres/spheroids (1, 2, 7, 49). Further, stem cells from other tissue sources are also often grown in suspension cultures to maintain stem cell properties (50, 51). Taken together, spheres/spheroids formation may provide stem cells the condition that mimics the thyroid microenvironment so-called “niche” that regulates activities of thyroid stem cells in culture (52, 53).

## Stem Cells in Thyroid Regeneration

Many organs when undergoing partial excision, the remnant follows compensatory growth involving cellular hypertrophy and/or hyperplasia (54). Notably, the liver exhibits a remarkable regenerative capacity after partial hepatectomy; the liver mass returns close to the original weight by 7 days (55). Thyroid gland is among the organs that exhibit hypertrophy as well as hyperplasia after partial thyroidectomy (29, 30) although without significant changes in its size (27). By analogy to partial hepatectomy, partial thyroidectomy may be used to study and understand the mechanisms of thyroid regeneration even though the gland does not recover its original size.

### Partial thyroidectomy

In the past decades, partial thyroidectomy was mainly used to study the effect of decreased levels of endogenous thyroid hormone or exogenously administered thyroid hormone on liver regeneration, enzymatic activities/functions, or the levels of thyroid hormone-regulated molecules in the brain, hypothalamus, pituitary, and liver (56–61). Other studies used partial thyroidectomy to carry out quantitative analysis of the thyroid function after inoculating thyroid cells into partial/total thyroidectomized rodents and their relation to neoplasms (31–33).

**Figure 1:**
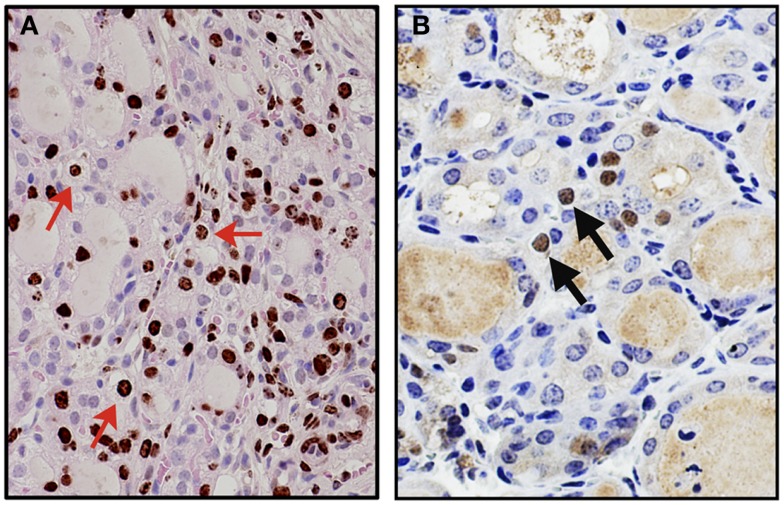
**Appearance of clear cells 2 weeks after partial thyroidectomy**. **(A)** Immunohistochemistry for BrdU (brown color) counterstained with light H&E. Many clear cells are positive for BrdU as representatives shown by red arrows. **(B)** Immunohistochemistry for Foxa2 (brown color), counterstained with hematoxylin. Clear cells expressing Foxa2 are indicated by arrows.

A mouse model for thyroid regeneration was established using partial thyroidectomy to study the effect of partial thyroidectomy on the morphology and gene expression of the remnant tissues/cells (62). In this study, partial thyroidectomy was carried out to remove one whole thyroid lobe and ~2/5 caudal segment of the other lobe. During this study, it was noted that the central areas of both thyroid lobes (without partial thyroidectomy) serve as the proliferative centers where many immature microfollicles, and bromodeoxyuridine (BrdU)-positive and/or C cells are located. One week after partial thyroidectomy, serum TSH levels were drastically elevated, resulting in a goitrogenesis condition, which was almost completely resolved by 2 weeks post-partial thyroidectomy. At that time, a marked increase of the number of BrdU-positive cells and cells with clear or faintly eosinophilic cytoplasm were observed in the central area and the area continuous to the cut edge (Figure 1A) (62). Some clear cells were BrdU-positive, suggestive of active proliferation, and expressed Foxa2, the definitive endoderm lineage marker (Figure 1B). Microarray followed by pathway analysis revealed that the expression of genes involved in embryonic development and cancer was affected by partial thyroidectomy. Electron microscopy demonstrated that clear cells were scant in cytoplasm, and retained features reminiscent of C cells having dense neuroendocrine granules or follicular cells juxtaposed to a lumen with microvilli. Taken together, clear cells may be immature cells that were previously C cells or follicular cells that participate in repair, regeneration of the thyroid gland, and/or goitrogenesis after partial thyroidectomy.

### Stem cell antigen-1 positive cells

The first evidence of participation of stem cell antigen-1 positive (SCA-1+) cells in development of thyroid follicles after partial thyroidectomy was provided by the use of β-galactosidase (β-gal) reporter mice in conjunction with partial thyroidectomy as a model for thyroid regeneration, and BrdU long label-retaining cell analysis (63). The β-gal reporter mice express β-gal in a thyroid follicular cell-specific fashion upon expression of Cre recombinase that is controlled by the human *TPO* gene promoter. The *TPO* promoter is regulated by NKX2-1 (10, 11), and becomes active around embryonic day (E) 14.5–15.5 of mouse gestation (64), around the time when thyroid hormone production commences (65). This indicates that cells expressing β-gal have gone through thyroid differentiation, thus β-gal mice being able to use in a follicular cell lineage tracing experiment.

**Figure 2:**
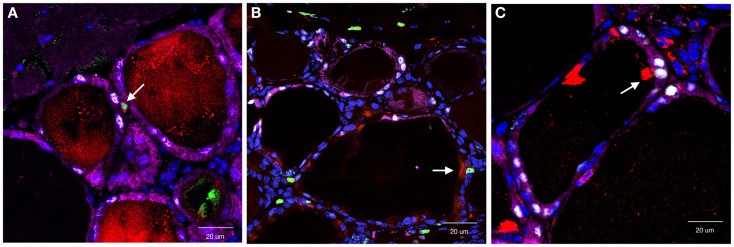
**Co-immunofluorescence for SCA1, BrdU, NKX2-1, and β-gal expression determined at day 7 (A), day 35 (B), and day 120 (C) post-partial thyroidectomy**. Colors used were red (SCA1), green (BrdU), white (NXK2-1), and purple (β-gal). At day 7, red and green co-stained positive cells [SCA1(+); BrdU(+)] were found in non-follicular mesenchymal area. At day 35, red/green positive signal was found as an intrafollicular cell, that did not express NKX2-1 (white), nor β-gal (purple). At day 120, intrafollicular cells expressing all four proteins were seen. Double or quadruple positive cell in each panel is shown by an arrow. In **(C)**, strong SCA1 signal is due to cilia that this SCA1(+) cell has.

In this study, the main focus was SCA1(+) cells based on the previous observation that SP cells exhibited stem/progenitor cell-like characteristics, and a half of them were SCA1(+) (34). Partial thyroidectomy was carried out by removing caudal one-third of both thyroid lobes as opposed to removing one whole thyroid lobe and ~2/5 caudal segment of the other lobe used in the study in which the presence of clear cells were observed (62). In non-partial thyroidectomized thyroid, SCA1(+) cells were occasionally found only in vascular endothelial cells. One to two weeks after partial thyroidectomy, SCA1(+)/BrdU(+), but β-gal(−)/NKX2-1(−) cells were found in the non-follicular mesenchymal areas (Figure 2A). They were negative for CD34, suggestive of non-hematopoietic origin, and also negative for OCT3/4, GATA4, and SOX10, a marker for stem cells, early endoderm cells, and neural crest-derived cells, respectively. However, lack of expression of these genes may have simply been due to missed timing for detection. Interestingly, they temporarily expressed cytokeratin 14 (KRT14), suggesting that they had started acquiring epithelial features. Some SCA1(+)/BrdU(+)/KRT14(+) cells were also found in part of follicles at 14 days post-partial thyroidectomy. After partial thyroidectomy, follicles in the area close to the cut edge became irregular in shape with low or no colloid inside, frequently having ciliated intrafollicular cells. The number of ciliated intrafollicular cells peaked at day 35 through 75 post-partial thyroidectomy. In the affected irregular follicles at day 35 post-partial thyroidectomy, most intrafollicular cells were SCA1(+)/BrdU(+)/β-gal(−)/NKX2-1(−), suggesting that these newly proliferated cells did not originate from previously differentiated follicular cells and have not yet differentiated to express NXK2-1 because NKX2-1 regulates *TPO* gene expression, on which β-gal expression depends (Figure 2B). However, at day 120 post-partial thyroidectomy thyroid, some intrafollicular cells were found to be SCA1(+)/BrdU(+)/β-gal(+)/NKX2-1(+) (Figure 2C). This suggests that after partial thyroidectomy, the irregular shaped follicles may become functional follicles expressing TPO. It was estimated that only ≤0.1% of intrafollicular cells expressed SCA1 at day 35 post-partial thyroidectomy that may have participated in the regeneration and/or differentiation of thyroid follicular cells after partial thyroidectomy. In this study, however, it was not possible to demonstrate the origin of SCA1(+) cells, whether SCA1(+) cells actually migrate from mesenchymal areas into follicles, and whether SCA1 can be used as a marker for thyroid stem/progenitor cells. The origin of SCA1(+) cells may be resident adult thyroid stem/progenitor cells, bone marrow-derived cells (see below), or cells derived from yet to be determined origins. That no CD34 expression were found in SCA1(+) cells suggests against that SCA1(+) cells are bone marrow-derived cells. However, the possibility cannot be excluded that bone marrow-derived cells have changed their characteristics after settling in the thyroid microenvironment that includes loss of CD34 expression. These questions remain to be answered.

### Bone marrow stem cells

It is known that bone marrow-derived mesenchymal stem cells are pluripotent progenitors that have self-renewal proliferation and multipotent differentiation capacity into many lineages of tissues from mesoderm, ectoderm, and endoderm origins, and they home in response to injury signals (66–68). The mesenchymal stem cells, however, exhibit high heterogeneity without expression of specific surface markers (66–68). GFP-expressing bone marrow cells derived from C57BL/6 mice were grafted to irradiated C57BL/6 mice to study whether mesenchymal stem cells participate in the thyroid gland regeneration after X-ray exposure (69). GFP-positive signals in cells which co-expressed TG were found in thyroid follicles at the rate of ~6% when examined at 40 weeks post-X-ray irradiation at 5 Gy. This was the first demonstration that bone marrow mesenchymal stem cells indeed participate in the repair of thyroid after irradiation.

### Experimental autoimmune thyroiditis model

In the experimental autoimmune thyroiditis model mice established by immunization with TG, their thyroids were virtually completely destroyed after a month (70). However, the thyroid showed remarkable regeneration when observed up to 100 days, suggesting the presence and activation of adult thyroid stem cells (70). On day 0 of immunization, the expression of *Oct4* was observed by RT-PCR, the level of which decreased in the thyroid of day 35 post-immunization, in keeping with the notion that OCT4 expression decreases when stem cells begin differentiation (71). Regeneration was faster in the absence of CD24, possibly due to the effect of CD24 on the infiltrating lymphocytes. These mice may provide an alternative model to study the mechanisms of thyroid regeneration.

## Solid Cell Nest

Solid cell nest (SCN) is the structure believed to be the embryonic remnant derived from the ultimobranchial body (UBB) (72). UBB is the caudal lateral outpocketing from the fourth pharyngeal pouches that fuses with thyroid primordium around E14.5 in mouse gestation, giving rise to calcitonin-producing C cells (73). SCNs were described as an atypical type of follicle characterized in rats and mice as having non-homogenous or foamy colloid with occasional presence of cilia (74–76). SCNs are in irregular structures showing squamoid, glandular, or microcystic features with occasional presence of cilia (77–80) and are located in the middle third of the thyroid lateral lobes in humans (81). SCNs are composed of two cell types; “main cells” of polygonal or elongated shape with abundant eosinophilic cytoplasm and round-to-oval nuclei, and “C cells.” Some SCNs contained so-called “mixed follicles,” the structure lined by main cells and follicular epithelium with colloid and/or clumps of eosinophilic materials in the lumen (77–80). SCNs are often found associated with Hashimoto’s thyroiditis, a subset of papillary thyroid carcinomas, and rare tumors such as mucoepidermoid and squamous carcinomas of thyroid (77–80, 82).

Studies using histopathological and immunohistochemical analyses demonstrated the expression of p63 in the main cells of SCNs in basal/stem cell patterns (78–80). p63 is a member of the p53 family of transcription factors, and is highly expressed in the proliferative basal cell layer of stratified epithelia, where epithelial progenitor cells are thought to reside, including the skin, breast, esophagus, cervix, urogenital tract, and prostate (83–87). p63 plays an important role in tumorigenesis, epidermal differentiation, and stem cell self-renewal (86, 87). In addition to p63, the main cells of SCNs express telomerase and bcl-2 (79), both of which are known to be associated with stem cells and cancer (88–90). p63 is a highly specific marker for SCNs, and its expression is frequently associated with Hashimoto’s thyroiditis and papillary thyroid carcinomas (78–80). Taken together, it was proposed that SCNs may represent a pool of stem cells that could contribute to the histogenesis of C cells and follicular cells, and to thyroid lesions as described above (77–80, 82).

### Presence of immature cells in SCN

**Figure 3:**
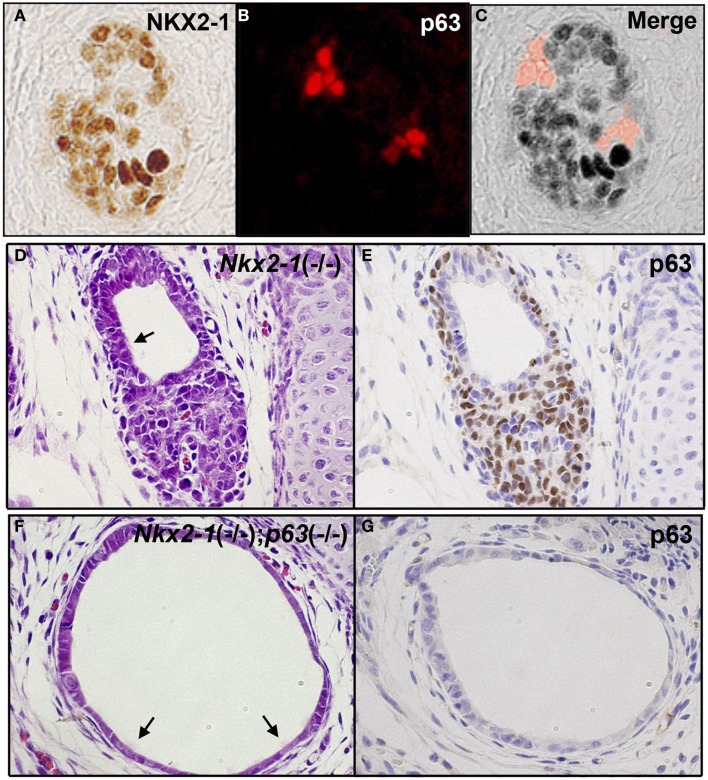
**p63 expression in UBB and SCN of thyroid**. **(A–C)** Immunofluorescence of E13.5 UBB of wild-type mouse embryos for NKX2-1 **(A)** and p63 immunohistochemistry **(B)**, and merged image **(C)**. Majority of cells express NKX2-1 while a few cells express p63, both being without overlap. **(D,E)** SCN from E18.5 *Nkx2-1*-null embryos for H&E **(D)** and p63 immunohistochemistry **(E)**. p63 is expressed in the stem/basal cell patterns. **(F,G)** SCN from E18.5 *Nkx2-1*; *p63*-double null embryos for H&E **(F)** and p63 immunohistochemistry **(G)**. Note that the monolayer of p63-negative cells remain in SCN from *Nkx2-1*; *p63*-double null embryos. Arrows indicate ciliated cells observed in the cystic structure.

Ultimobranchial body is composed of two types of cells: one expressing NKX2-1 and the other expressing p63 (91). Most cells express NKX2-1 while only a few cells express p63 when examined at E13.5, the time before UBB fuses with the thyroid primordium (Figures 3A–C) (91, 92). The NKX2-1-expressing cells do not overlap with the p63-expressing cells. Using *Nkx2-1*-null mice (8), it was demonstrated that NKX2-1-expressing cells were essential for survival of the UBB cells during its migration to meet with thyroid primordium, while p63-positive cells could proliferate in the absence of NKX2-1 expression (91). At E18.5, the latter cells gave rise to a mass of p63-positive cells in a nested pattern and/or a cystic vesicular structure that was lined by a monolayer of p63-negative cells of unknown phenotype, surrounded by a cluster and/or single layer of p63-positive cells, displaying the basal/stem cell appearance (Figures 3D,E). Occasionally, ciliated cells were found in the lining epithelium of the vesicle (Figures 3D,F). This structure demonstrated a striking similarity in appearance to SCN. Note that the thyroid primordium cells of *Nkx2-1*-null mice disintegrated through apoptosis (93) and only this cystic structure remained in *Nkx2-1*-null mice around the area where the thyroid was supposed to be located (91, 92). On the other hand, p63 was not required for the normal development of thyroid gland (92).

The nature of the cystic structure found in *Nkx2-1*-null mice was further studied using *Nkx2-1*; *p63*-double null mice (92). In these mice, the p63-positive outer layer of cells surrounding the cystic vesicular structure or those in nested patterns continued to the vesicular structure were no longer found. Instead, only p63-negative single layer cells in tubular or cystic patterns remained, which appeared dilated (Figures 3F,G) (92). Occasionally, ciliated cells were observed in the cysts (Figures 3F,G) (92). Cells of the *Nkx2-1*; *p63*-double null UBB cysts were negative for all the following markers; neuron-specific enolase and S100 as neuron-specific markers, OCT4 as a stem cell marker, nestin and SOX10 as a neural crest marker, and vimentin as a mesenchymal marker. Electron microscopic examination revealed that the *Nkx2-1*; *p63*-double null UBB were composed of immature cells with scarce cytoplasm and no clear intracellular organelles or adhesion structures (92). While the presence of these immature cells were observed only in *Nkx2-1*; *p63*-double null mice, they may always be present within the thyroid of wild-type mice; whether they are dispersed as individual cells throughout the thyroid, or stay clustered within thyroid remain unknown.

In the partial thyroidectomy model mice, ciliated intrafollicular cells were mainly found in the area where many irregular shaped follicles were present, which drastically increased in number after partial thyroidectomy; 1 in 100 intrafollicular cells had cilia (63), which otherwise were only sporadically found in fully developed adult thyroids (78–80). Further, in these irregular follicles, more number of newly differentiated intrafollicular cells was found after partial thyroidectomy (63). It is possible that the immature cells found in SCN may represent irregular shaped follicles with ciliated intrafollicular cells that become functional follicles after partial thyroidectomy. However, these cells do not express OCT4, suggesting that they may be different from OCT4-expressing adult-resident thyroid stem/progenitor cells.

## Models for Thyroid Regeneration

**Figure 4:**
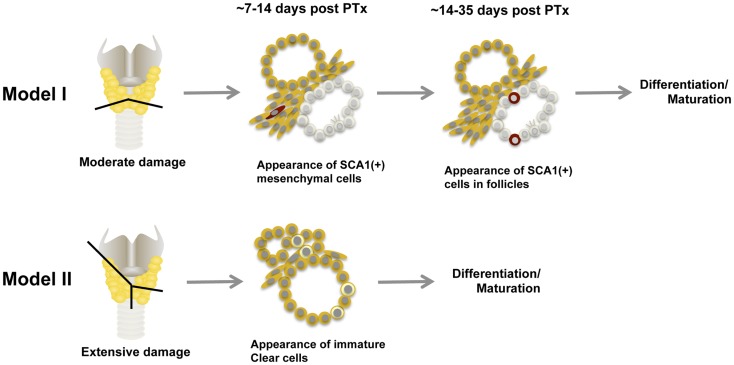
**Two models for thyroid regeneration in mice**. Model I may be operable when the damage to the thyroid is modest such as when a small portion of thyroid (tissues below the line) is removed. This model may take 2–4 weeks to start producing functionally matured thyrocytes, and may involve immature cells in SCN. SCA1-expressing cells and SCN-like immature cells are shown in red and in pale color, respectively. Ciliated cells are also shown within the immature cells. Model II may become functional when the thyroid damage is massive such as semi-total partial thyroidectomy (a whole lobe and the other lobe below the line as indicated are removed). In this case, follicular cells and C cells are reset to become clear immature cells (shown in light yellow) to start the maturation process again. This model may take 1–2 weeks to start producing functionally mature thyrocytes. Both models can operate simultaneously. PTx, partial thyroidectomy.

Based on what is described above, at least two models for thyroid regeneration can be proposed, particularly when the damage is caused by partial thyroidectomy (Figure 4) (63). Model I: SCA1(+) newly synthesized [BrdU(+)] non-follicular mesenchymal cells appear after partial thyroidectomy, followed by appearance of SCA1(+); BrdU(+) intrafollicular cells, which eventually become part of functional follicles. The SCN-like immature cells that are dormant under normal circumstances, may become active to collaborate with SCA1(+) cells to participate in new follicle formation. How this happens and whether a newly formed follicle further participates in formation of additional follicles remains unknown. SCA1(+) cells may be resident adult thyroid stem/progenitor cells that have lost OCT4 expression and acquire SCA1 expression upon stimulation by partial thyroidectomy. Alternatively, SCA1(+) cells could be bone marrow-derived cells that have changed their characteristics after becoming residents in the thyroid; after partial thyroidectomy, they are activated to participate in thyroid regeneration. There could be other mechanisms for the appearance of SCA1(+) cells that are currently unknown. Model I is operative in a situation where thyroid damage caused by partial thyroidectomy is moderate, such as removing the caudal one-third of both thyroid lobes, and takes 2–4 weeks to produce new intrafollicular cells. Model II kicks in when the damage is massive, exceeds the capacity repaired by Model I, and requires immediate repair to maintain body homeostasis and correct goitrogenesis condition. This is the case when the thyroid underwent semi-total partial thyroidectomy (one lobe and 2/5 of the other thyroid lobe removed), in which previously differentiated follicular cells and C cells appear to become immature cells and start contributing thyroid regeneration. This model takes place within 1–2 weeks. Perhaps resetting many cells to the immature stage may make the differentiation/maturation proceed more robustly, resulting in more rapid restoration of thyroid function. These two thyroid regeneration models somewhat resemble to those of liver regeneration where one model involves mature liver epithelial cells (i.e., hepatocytes and cholangiocytes) and the other involving liver stem/progenitor cells (94–97). The two thyroid regeneration models may be operable in other damaged situations than partial thyroidectomy where thyroid requires repair. They may take place individually or simultaneously, depending on the extent of injury and regeneration required. Based on our experience, some clear cells were found in moderately partial thyroidectomized thyroid (Model I), suggesting that the Model II pathway may always be activated as a supplemental repair mechanism.

## Conclusion

We have begun understanding the nature of adult thyroid stem/progenitor cells and possible mechanisms of their involvement in thyroid regeneration when thyroid is damaged and requires repair. At least two models for thyroid repair are proposed involving different types of stem/progenitor cells; one for mature cells reprogramed back to immature endoderm lineage-committed progenitor cells, and the other involving cells that appear to be adult-resident stem/progenitor cells. Processes required for tissue repair may have many similarities to cancer development and metastasis, or may provide insight into the development of various other thyroid diseases. Understanding normal adult thyroid stem/progenitor cells and their involvement in thyroid repair should help better understand these diseases, hopefully leading to better diagnosis and/or treatment.

## Conflict of Interest Statement

The author declares that the research was conducted in the absence of any commercial or financial relationships that could be construed as a potential conflict of interest.
